# SCMMTP: identifying and characterizing membrane transport proteins using propensity scores of dipeptides

**DOI:** 10.1186/1471-2164-16-S12-S6

**Published:** 2015-12-09

**Authors:** Yi-Fan Liou, Tamara Vasylenko, Chia-Lun Yeh, Wei-Chun Lin, Shih-Hsiang Chiu, Phasit Charoenkwan, Li-Sun Shu, Shinn-Ying Ho, Hui-Ling Huang

**Affiliations:** 1 Institute of Bioinformatics and Systems Biology, National Chiao Tung University, Hsinchu, Taiwan; 2 Department of Biological Science and Technology, National Chiao Tung University, Hsinchu, Taiwan; 3 Department of Information Management, Overseas Chinese University, Taichung, Taiwan; 4 Center for Bioinformatics Research, National Chiao Tung University, Hsinchu City, Taiwan

**Keywords:** Membrane transport protein, dipeptide composition, physicochemical property, scoring card method, support vector machine

## Abstract

**Background:**

Identifying putative membrane transport proteins (MTPs) and understanding the transport mechanisms involved remain important challenges for the advancement of structural and functional genomics. However, the transporter characters are mainly acquired from MTP crystal structures which are hard to crystalize. Therefore, it is desirable to develop bioinformatics tools for the effective large-scale analysis of available sequences to identify novel transporters and characterize such transporters.

**Results:**

This work proposes a novel method (SCMMTP) based on the scoring card method (SCM) using dipeptide composition to identify and characterize MTPs from an existing dataset containing 900 MTPs and 660 non-MTPs which are separated into a training dataset consisting 1,380 proteins and an independent dataset consisting 180 proteins. The SCMMTP produced estimating propensity scores for amino acids and dipeptides as MTPs. The SCMMTP training and test accuracy levels respectively reached 83.81% and 76.11%. The test accuracy of support vector machine (SVM) using a complicated classification method with a low possibility for biological interpretation and position-specific substitution matrix (PSSM) as a protein feature is 80.56%, thus SCMMTP is comparable to SVM-PSSM. To identify MTPs, SCMMTP is applied to three datasets including: 1) human transmembrane proteins, 2) a photosynthetic protein dataset, and 3) a human protein database. MTPs showing α-helix rich structure is agreed with previous studies. The MTPs used residues with low hydration energy. It is hypothesized that, after filtering substrates, the hydrated water molecules need to be released from the pore regions.

**Conclusions:**

SCMMTP yields estimating propensity scores for amino acids and dipeptides as MTPs, which can be used to identify novel MTPs and characterize transport mechanisms for use in further experiments.

**Availability:**

http://iclab.life.nctu.edu.tw/iclab_webtools/SCMMTP/

## Background

Membrane transport proteins (MTPs), or transporters, span lipid bilayers and form gates for hydrophilic solutes to cross hydrophobic membranes. Transporters are essential in many biological processes, such as nutrient uptake, metabolite secretion, ion homeostasis, signaling, energy transduction, immune system recognition processes, osmoregulation, and other physiological and developmental processes in the cell [1]. Currently, several commercial drugs target ion channels or carrier proteins [2]with results indicating that transporter proteins have tremendous therapeutic potential [3,4].

MTPs are primarily involved in the transportation of amino acids, cations, anions, sugars, proteins, mRNAs, electrons, water, and hormones [1]. According to the transporter nomenclature panel of the International Union of Biochemistry and Molecular Biology, MTPs can be classified into six groups based on their mode of transport, energy coupling mechanisms, molecular phylogeny, and substrate specificity [3]. MTPs are thought to constitute 3-16% of the total number of open reading frames in prokaryotic genomes [5]. Identifying putative MTPs and understanding their transport characters are important challenges in the advancement of structural and functional genomics. MTPs have been identified by proteomics strategies, such as absorbance spectroscopy, gel electrophoresis, metal-affinity columns and shift assay, chromatography, mass spectroscopy, and combined spectroscopic studies [6]. But two main features of the MTP make them difficult to identify [7]. First, transporters are usually minor components in cell membranes. The protein engineers often use *E. coli *or yeasts as the hosts to overexpress MTPs which seems to be toxic after overexpressing or expressed as unfolded inactive proteins [7]. Second, most MTP contain a series of hydrophobic residues causing the under-represented in two-dimensional electrophoresis [7]. Bioinformatics tools are needed for effective large-scale analysis of available sequences to identify novel transporters, direct further experiments and provide information about transport mechanisms.

Recently, several machine learning methods have been proposed for predicting membrane transporters from amino acid sequence information. Lin et al. [4] used a support vector machine (SVM) to predict transporter families from the transporter classification system. Gromiha et al. [8] analyzed the amino acid compositions in MTPs and used different classifiers implemented in the WEKA program to discriminate channel/pore proteins, electrochemical transporters, and active transporters. Li et al. [9] developed a general approach combining homology-based and machine learning methods, using transporter sequence features learned from well-curated proteomes as guides, to predict major transporter families/subfamilies defined in the transporter classification database. Ou et al.[10] analyzed the amino acid composition of transporters and developed a radial basis network-based method for classifying these proteins into channel/pore proteins, electrochemical transporters, active transporters, and six transporter families with amino acid properties and position-specific substitution matrix (PSSM) profiles. Mishra et al. [1] contributed to the substrate specificity annotations of transporters by developing SVM models that discriminate between amino acid, anion, cation, electron, protein/mRNA, sugar, and other transporters. The transporter characters can be investigated based on the crystal structure of the transport proteins and their transport objects. Sauguest et al. [11] used pentameric ligand-gate ion channels to examine ion permeation. Hibbs and Gouaux[12]identified permeation and activation principles in an anion receptor. Zhou et al. [13] and Kopfer et al. [14] used potassium channels to determine the relationship between ion coordination and hydration, as well as the Coulomb knock-on mechanism. However, the goal of most studies has been to predict channel families; a few studies have constructed a general predictor to predict if the proteins are channel proteins. Although these predictors can provide a range of prediction accuracy levels, independent statistical work is needed to examine MTP characters, whose understanding is mostly based on crystal structures and also to some extent on sequences.

**Figure 1:**
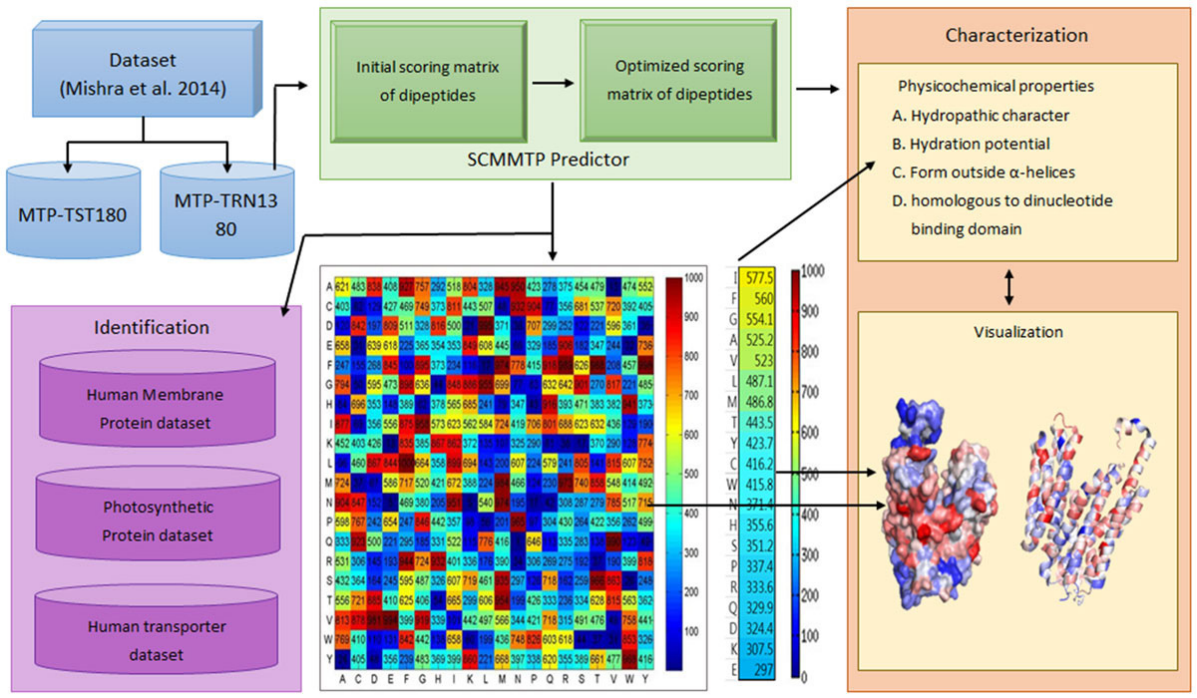
**The flowchart for identification and characterization of MTP**. The generated amino acids and dipeptides scoring cards from SCMMTP are used for MTP identification and analysis.

Here, we propose an approach based on the Scoring Card Method (SCM; referred to as SCMMTP), which uses dipeptide composition as a feature for predicting and characterizing MTPs. As shown in Figure 1, the existing dataset [1] is separated into test and training datasets. The training dataset is used to build the scorecard for prediction, visualization, and analysis to obtain new information.

SCM can provide insight into protein function prediction based on interpretable propensity scores [15-18]. To create the SCMMTP, a previously published dataset [1] was used. The proposed method estimated the propensity scores of 400 individual dipeptides and used the difference between dipeptide compositions of positives and negatives to predict putative transporters. The method was further optimized using an Intelligent Genetic Algorithm (IGA) [19]. The propensity scores of 20 natural amino acids were derived from the dipeptide scores and used to identify informative physicochemical properties (PCPs) of membrane transporters. The SCM method achieved a 10-fold cross validation accuracy (10-CV) of 81.12% and a test accuracy of 76.11%. Several PCPs from the AAindex database [20] or from some PCP studies have been useful for describing transporters. First, the "hydropathy index" scale (KYTJ820101) was found to precisely reflect the conformational characteristics of transporters. Second, MTPs are expected to have higher preferences for α-helices outside, rather than inside, of protein molecules (WERD780104). Finally, the channels are generally composed of residues with low hydration energy levels. This occurs because after hydrated solutes pass through the membrane via MTPs, the channel must release the water molecules so that additional substances can be transported.

## Materials and methods

In this work, we propose a novel SCMMTP for the identification and characterization of MTPs based on the propensity scores of dipeptides and amino acids. The MTP characterization includes the analysis of protein PCPs, the visualization of the MTP propensity scores and a PCP mining method. This methodutilized the propensity scores of amino acids allowing for the analysis of the MTPs. Figure 1 presents a flowchart of the experimental design.

### Dataset

We established five datasets based on different sources of transporter proteins from various species: MTP-TRN1380, MTP-TST180, HTS380, HMTP494 and PSPGO649. MTP-TRN1380 and MTP-TST180 were respectively used as training data for the SCMMTP classifier and independent test. HTS380 and HMTP4942 both contained human transporter proteins. PSPGO was composed of the photosynthetic proteins. HTS380, HMPAS4942 and PSPGO were used for the identification of MTPs. The numbers of transporters and non-transporters in each dataset are summarized in Additional File 1: Table S1.

#### MTP-TRN1380 and MTP-TST180

Mishra et al. [1] provided a dataset which included 10,780 transporter, carrier, and channel proteins collected from the UniProt database. Mishra et al. removed fragmented sequences and sequences annotated with more than two substrates, those based solely on sequence similarity, and sequences which exhibited a similarity exceeding 70%. The primary dataset contained 900 transporters and 660 non-transporters that were randomly chosen from all proteins in UniProt, excluding 10,780 MTPs for the negative dataset. The 1,560 sequences in our dataset were divided into training and test datasets. The training dataset, named MTP-TRN1380, consisted of 780 transporters and 600 non-transporters, while the test dataset, named MTP-TST180, included 180 transporters and 60 non-transporters.

#### HTS380

Huang et al. [21] gathered 5,176 human transporter proteins from SwissProt. Huang et al. divided the sequences into"confirmed", "potential" and "non-transporter" by manually checking four annotations in SwissProt, i.e., protein names, gene names, function and sequence similarities. After reducing the sequence identity with a threshold of 25%, HTS had 380 transporters, 144 potential transporters and 2,815 non-transporters. The 380 transporters, named HTS380, are used for the identification of MTPs.

#### HMPAS4942

Kim and Yi [22] built a human protein database containing36,585 proteins with 5386 transporters. This study considered the transporters members from this database, excluding the sequences having uncommon amino acids. Finally, 4,942 transporters were collected as HPMAS4942.

#### PSPGO649

Most photosynthetic proteins are membrane-embedded and take part in electron transport reactions of photosynthesis. This process involves the transport of electrons, protons and other solutes via proton complex. This work adopted the PSPGO dataset from the previous study [23] to identify MTPs. The sequence identity was reduced to 25%. PSPGO contained 649 photosynthetic proteins as positive dataset and 649 randomly chosen sequences from non-photosynthetic proteins as the negative dataset. In this work, we used only positive part of PSPGO, called PSPGO649.

### SCM-based MTP classifier (SCMMTP)

The Scoring Card Method (SCM) was already used to analyze various protein functions [15-18] from sequence information. In contrast to the SVM classifier, SCM demonstrates increased simplicity and interpretability by using the propensity scores of amino acids and dipeptides to identify and characterize protein function. Current work proposes the SCM-based method (SCMMTP) to predict MTPs. The SCMMTP implementation corresponds to the original SCM algorithm without any major adjustments, as follows:

Construct a training dataset, MTP-TRN1380, consisting of 780 MTPs and 600 non-MTPs.

Calculate the normalized dipeptide propensity scores of the MTPs in therange from 0-1000. According to Huang et al [16], the dipeptide compositions are calculated as follows:

(1) $$\text{DPC}\left(i,j\right)=\frac{N_{ij}}{L-1},1\leq i,j\leq 20$$

where *i,j *indicate the distribution of amino acid *i *followed by amino acid *j*, N denotes the dipeptide numbers of the dipeptide composed with amino acid *i *followed by amino acid *j*, and *L *is the total residue numbers of the sequences. These scores revealed the dipeptide compositions of the MTPs minus non-MTPs. The propensity score of each amino acid × was then easily computed by averaging all dipeptides containing this amino acid X. Finally, the normalized scores were generated as follows:

(2) $$S_{i}^{\prime }=\frac{1000\left(S_{i}-Min_{i}\right)}{Max_{i}-Min_{i}}$$

where i indicates the residues.*S'_i_*, *S_i _*, *Max_i _*and *Min_i _*denote the scaled target dipeptide compositions, original target dipeptide compositions, maximum dipeptide compositions and minimum dipeptide compositions, respectively, of the corresponding residues.

Use IGA to optimize the dipeptide propensity scores (DPS) in order to maximize the prediction accuracy and to conserve the original sequence information. The fitness function of IGA is concerned with both the area under the ROC curve (AUC) [24] and the Pearson's correlation coefficient (i.e., the R value) between the initial and optimized propensity scores of 20 amino acids. The weights for the AUC and R value were set based on the previous studies [15-18,23]. (See Eq. 1).

(3) maxFit(DPS)=0.9×AUC+0.1×R

Create MTP predictor by defining a scoring function *S(P)*in which *P *is a query protein sequence, and and are respectively the composition and propensity score of the dipeptides *i*. The threshold is an optimal score separating the MTPs and non-MTPs in MTP-TRN1380. If *S(P) *is greater than the threshold value *P *is MTP, otherwise *P *is a non-MTP.

(4) $$S\left(P\right)=\overset{400}{\underset{i=1}{\sum }}w_{i}DPS_{i}$$

### Generic-MTP classifiers

The SCMMTP performance was compared toother classifiers with the features commonly used in protein function prediction. This work considered the SVM, J48, Bayes and k-Nearest Neighbor (KNN) in cooperation with theamino acid composition (AAC), the dipeptide composition (DPC), the normalized PSSM (PSSM400) [25], and the 531 PCPs fromthe AAindexdatabase as the features. SVM is widely applied for protein function prediction and is also implemented for MTP classification [1]. We used LIBSVM [26] to create SVM classifiers with radial basis kernel. The optimal SVM parameters were chosen via a grid search according to the 10-fold cross-validation (10-CV) accuracy of MTP-TRN1380. Other classifiers are implemented using WEKA [27]. The suitable K parameter of the KNN classifier was decided based on the best 10-CV evaluated from MTP-TRN1380. We tried 5 different K values for each KNN classifiers i.e. 3, 5, 7, 9 and 13. We used the default WEKA parameter settings when applying both the decision tree (J48) and the Naïve Bayes classifiers.

### MTP characterization

MTPswere analysed and characterized using the PCP mining method and the propensity score visualization method.

The PCP mining method, SCM-PCPs, was introduced to identify the important physicochemical properties (PCPs) of Heme-binding proteinsbased on the propensity scores of 20 amino acids [17]. To find a set of possibly correlated PCPs with a considered protein function, we examined the 544 indices representing different PCPs from the AAindex database. After removingthe PCPs containing the value 'NA', 531 PCP indices were left and considered in this work. The implementation of SCM-PCPs to MTPs analysis included following steps: 1) Calculate the R values between the amino acid propensity scores of MTPs (generated by SCMMTP) for each of the531 PCPs. 2) Calculate the R values between the amino acid propensity scores and the informative PCPs collected based on the domain knowledge of MTPs. 3) If the R values of the PCP and amino acid propensity scores of MTP > 0.5, these PCPs are chosen as candidate PCPs for further analysis.

The visualizing method aimed to express the MTP propensity scores for proteins to determine their characteristics. The structure coordination files of the proteins werecolored according to the amino acid or dipeptide scores, and expressed using Pymol [28]. The red and blue colorsrespectively represented high and low propensity score residues.

## Results and discussion

### Performance comparisons of different MTP predictors

Because of the variance in the MTP datasets, many predictors used different MTP datasets for creating their predicting models. For example, TPpred [6] used the mitochondrial proteins as the dataset while TransportTP used sequences from the TCDB database [29]. We evaluated the performance of the SCMMTP method and other generic-MTP classifiers (Decision tree, J48; Naïve Bayes; K-nearest neighbors, KNN; Support vector machine, SVM) with four types of feature sets (amino acid composition, AAC; dipeptide composition, DPC; physicochemical property, PCP; Position-Specific Scoring Matrix, PSSM) to discriminate between MTPs and non-MTPs.

The SCMMTP uses IGA, a nondeterministic method, meaning that the result of each run is independent. Therefore, 10 independent runs of SCMMTP on a training set were evaluated, with the results presented in Additional File 2: Table S2. The first experiment 1_st _exhibited the highest training accuracy (81.71%) and was selected for further analysis. The sensitivity, specificity, and a threshold value for this model were 85%, 78%, and 476.88, respectively. The performance of SCMMTP and other machine learning methods on MTP-TST1380 and MTP-TRN1380 are respectively shown in Table 1 and Additional File 3: Table S3.

**Table 1:** Performance comparison of MTP predictors on the independent test set

Method	Acc (%)	Sensitivity (%)	Specificity (%)	MCC
Bayes-AAC	50.00	28.33	93.33	0.25
Bayes-DPC	44.44	23.33	86.67	0.12
Bayes-AAindex	73.33	80.00	60.00	0.40
Bayes-PSSM	65.00	70.83	53.33	0.24
J48-AAC	66.67	58.33	83.33	0.40
J48-DPC	59.44	57.50	63.33	0.20
J48-AAindex	69.44	75.00	58.33	0.33
J48-PSSM	69.44	78.33	51.67	0.30
KNN-AAC(k = 7)	71.67	72.50	70.00	0.41
KNN-DPC (k = 5)	69.44	81.67	45.00	0.28
KNN-AAindex (k = 7)	75.00	82.50	60.00	0.43
KNN-PSSM (k = 13)	76.67	89.17	51.67	0.45
SVM-AAC	61.67	91.80	46.22	0.38
SVM-DPC	70.56	93.51	53.40	0.49
SVM-AAindex	72.78	78.33	61.67	0.40
SVM-PSSM	80.56	85.12	71.19	0.56
SCMMTP	76.11	80.00	68.33	0.47
Mean	67.78	72.13	63.38	0.36

The SCMMTP yielded test accuracy, sensitivity, specificity and MCC results of 76.11%, 80.00%, 68.33% and 0.47, respectively. SCMMTP had better performance than other predictors excluding KNN-PSSM and SVM-PSSM which showed the accuracies of 76.67% and 80.56%, respectively. Even the predictor using PSSM as thefeatures also had a good performance to predict the MTPs in previous study [1], this feature cannot always provide satisfied performances. The predictors, Bayes-PSSM and J48-PSSM, had only the accuracies of 65.00% and 69.44%, respectively. Among the predictors using DPC as feature, SCMMTP had a better performance than Bayes-DPC, J48-DPC, KNN-DPC and SVM-DPC which have the accuracies of 44.44%, 59.44%, 69.44% and 70.56%, respectively. The classifiers using AAC as feature generally had low performances excluding the KNN-AAC which had the accuracy of 71.67%. TheBayes-AAC, J48-AAC and SVM-AACshowedthe averaged accuracyof 59.48% with the Bayes-AAC yielding the lowest performance among all the predictors. This suggests that AAC would not be a good feature to predict MTPs even using different machine learning methods.

The SVM-PSSM method outperformed other classifiers with a test accuracy of 80.56%. However, the SVM uses a complicated classification model with a low possibility for biological interpretation. Moreover, the time cost issue is also a problem, while generating a PSSM profile takes a long time. On the other hand, SCMMTP with a straightforward weighted-sum model and a dipeptide composition as a feature set provides propensity scores which are interpretable in biological analysis.

### SCMMTP performance for identifying MTPs using existing datasets

The SCMMTP was used to identify MTPs of HMPAS4942, PSPGO649 and HTS380. The minimum and maximum scores, sensitivity and the number of identified MTPs are provided in Table 2. The sensitivities of HMPAS4942, PSPGO649 and HTS380 are 66%, 72% and 70%, respectively.

**Table 2:** Putative MTPs from Human membrane and Photosynthetic datasets

	**HMPAS4942**[22]	**PSPGO649**[23]	**HTS380**[21]
Total	4942	649	380
Transporter	3256	466	266
Non_ transporter	1686	183	114
Min score	321.89	398.33	428.06
Max score	601.50	636.15	547.37
Sensitivity	0.66	0.72	0.70

Errors in this MTP identification may be due to the different methodsused to establish the various datasets. In HMPAS4942, Kim and Yi [22] collected transporter sequences including both experimentally verified and predicted MTPs. The ten-lowest scoring sequences in our identification work do not have experimental evidence to be MTPs. These sequences are all predictedas MTPs using other prediction tools. Eight are predicted as MTPs (i.e., Q86XP4, Q9H480, Q6LA62, B4YCR0, H9PSV8, Q8HNQ6, Q658P4 and D6RA35, with respective scores of 321.89, 354.36, 354.56, 373.43, 375.93, 377.04, 377.32, and 379.32). In addition, Q8IYB3 and Q15287 (serine/arginine repetitive matrix protein 1, 364.88 & RNA-binding protein with serine-rich domain 1, 383.91) are selected using the ortholog selection methods due to a lack of experimental evidence.

The PSPGO649 dataset contained photosynthetic proteins, which are very diverse in terms of structure and function, ranging from soluble to membrane-embedded. Furthermore, membrane photosynthetic proteins do not necessarily traverse the bilayer, as they often are subunits of bigger protein complexes. Thus, the PSPGO649 dataset is expected to contain many MTPs, but is not completely a membrane-transporter dataset. Those proteins classified by SCMMTP as negative may be fragments or subunits of larger photosynthetic proteins, auxiliary subunits, soluble proteins of the Calvin Cycle, components of light-harvesting complexes, or proteins that help to activate other photosynthetic proteins. In addition, a closer look at the bottom-10 sequences shows that most of these sequences have homology-based annotations, which are not experimentally verified. On the contrary, sequences that have annotations based on experimental evidence (i.e., Uniprot ID P84990, P73202, and P09927) function as light receptors which does not imply a transporter function (see Table S4) [30,31].

The MTPs of HTS380 have characteristics similar to those in the MTP-TRN1380 and MTP-TST180 datasets. However, the sequences from HTS380 are only collected from humans, in whom some pore-forming proteins have functions which differ from those of the transporters. For example, Peroxisomal membrane protein 2 has score of 429.33 and is top-7 lowest scored sequences; it seems to be involved in pore-forming activity and may contribute to the unspecific permeability of the Peroxisomal membrane. This function is different from MTP-TRN1380 and MTP-TST180, in which the MTPs are permeable for specific subtracts.

### Comparing the dipeptide compositions of the MTPs and non-MTPs

The SCMMTP dipeptide scores revealed that the top-5 dipeptides with the highest scoresare LF, FY, DL, VE, and QV scored 1000, 998, 995, 994, and 990, respectively. The five dipeptides with the lowest scores were QN, NE, NK, FL, and AVwiththe scores of 1, 5, 5, 12, and 13, respectively. The averaged dipeptide compositions of MTP and non-MTP were calculated for comparison. Mann-Whitney U-test which is a non-parameter statistic method was applied to evaluate the statistical significance of averaged dipeptide compositions between MTP and non-MTP. In the top-5 ranked dipeptides, LF, DL, VE, and QV showed the significant differences based on a p-value threshold of 0.05, and had p-values of 0.00, 0.00, 0.01 and 0.03, respectively. However, FY had a p-value of 0.25 which was not significantly different between MTPs and non-MTPs. Among the lowest-5 scored dipeptides, QN, NE, NK, FL, and AV had the p-values of 0.00, 0.00, 0.00, 0.01 and 0.00, respectively, indicating a significant different between MTPs and non-MTPs. These results suggest that although the dipeptides with the highest and the lowest scores separated the MTPs and non-MTPs, some dipeptides that can be used after tuning these scores.

### MTPs characterization using the propensity visualizing method

The SCMMTP predictor operates by calculating dipeptide and amino acid propensity scores of MTPs and non-MTPs. Visualization techniques provide a way to represent these results and discover informative patterns within the structure of a given protein class. In this study, the protein structures were colored according to the SCMMTP-derived dipeptide (DP) and amino acid (AA) propensity scores.

**Figure 2:**
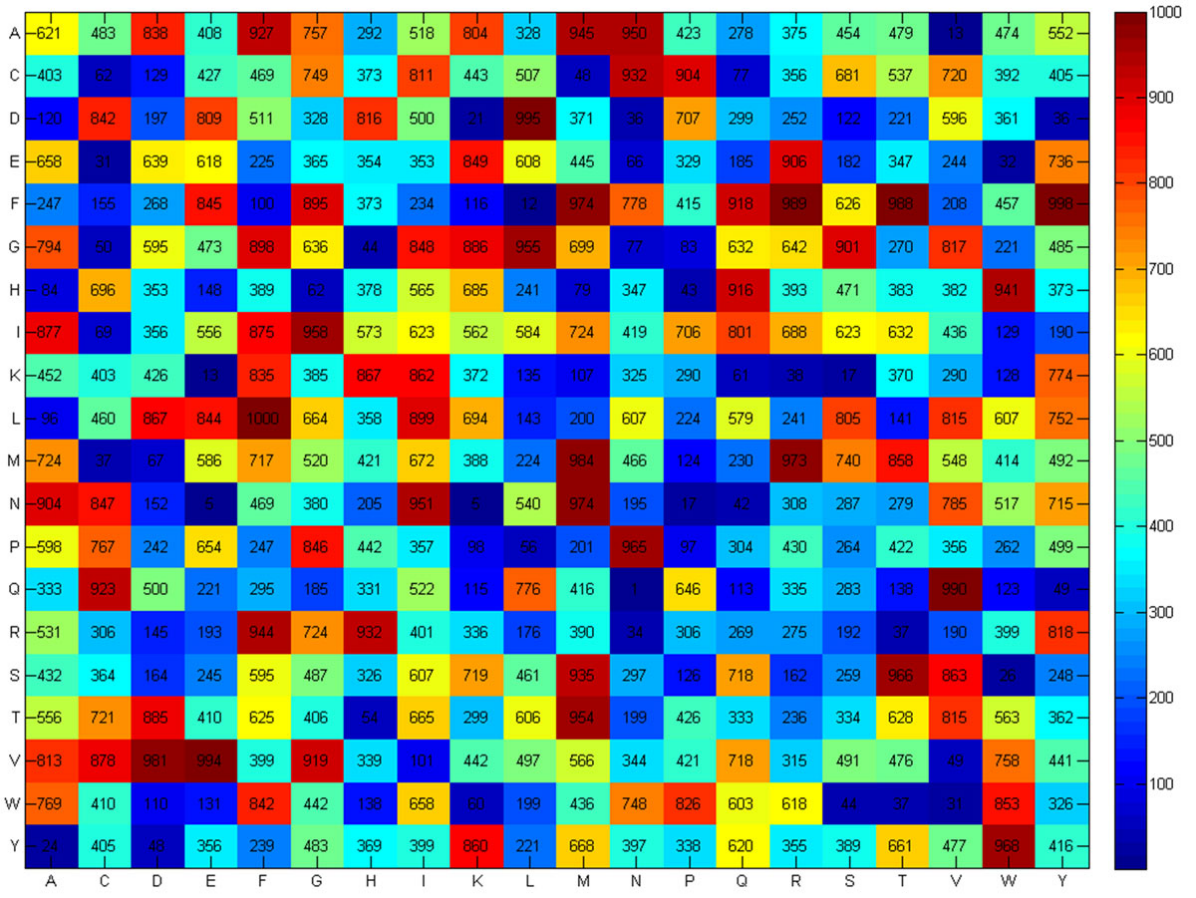
**Heat map of the dipeptide propensity scores generated by SCMMTP**.

**Figure 3:**
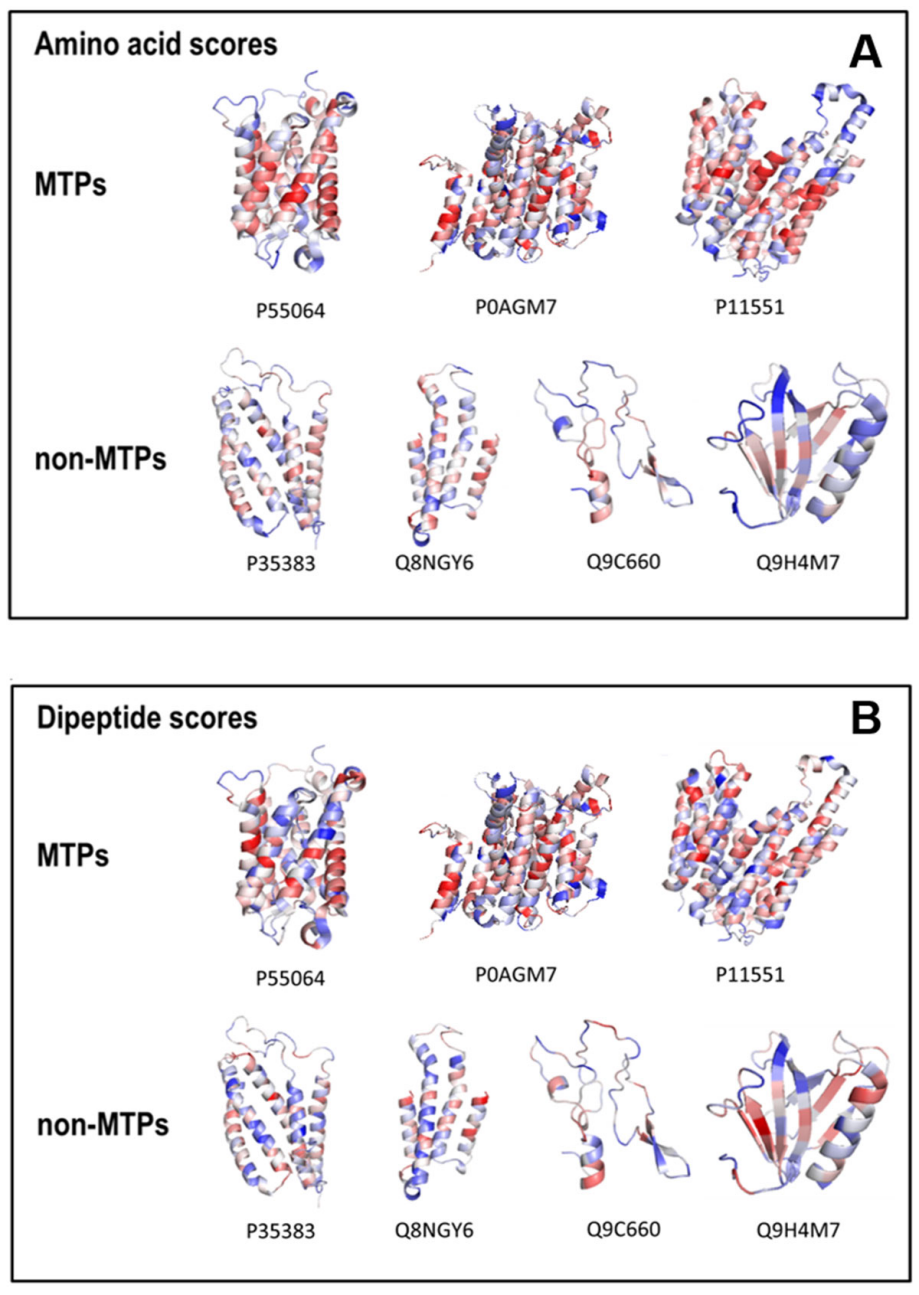
**The propensity score visualization of MTP**. A) SCMMTP dipeptide (DP) propensity scores visualization. B) SCMMTP amino acid(AA) propensity scores visualization. The MTPs Uniprot entries from the left are P55064, P0AGM7, and P11551; the non-MTPs PDB entries from the left are P35383, P49683, Q8NGY6, Q9C660, and Q9H4M7.

Figure 2 shows a heat map of the SCMMTP-derived propensity scores of 400 dipeptides as MTPs and non-MTPs. Five top-ranked dipeptides include LF, FY, DL, VE, and QV with respective scores of 1000, 998, 995, 994, and 990. Five dipeptides with the lowest scores are QN, NE, NK, FL, and AV, respectively scored 1, 5, 5, 12, and 13. The distributions of DP propensity scores on the surface of several highly-scored MTPs and non-MTPs have been visualized in Figure 3A. ThreeMTPs, P55064, P0AGM7 and P11551, respectively scored 498.2, 540.59 and 519.6, are selected, along with four non-MTPs, P35383, Q8NGY6M, Q9C660 and Q9H4M7, respectively scored 474.7, 451.5, 406.7 and 431.7. The red color represents the positions of highly-scored dipeptides, in contrast to the low-scored DP, which are colored in blue. Thus, MTPs contain more regions colored in red than non-MTPs.

Table 3 presents the propensity scores of 20 amino acids derived from the SCMMTP dipeptide scores and the amino acid compositions in MTPs and non-MTPs. Our results show that SCMMTP propensities are partially reflected in amino acid compositions of MTPs and non-MTPs. In particular, the residues Ile, Phe, and Gly, which are top-3 SCMMTP ranked, also have a composition difference greater than one (|Difference| > 1) among MTPs and non-MTPs. Notably Ile, Phe and Gly are hydrophobic and are dominant in MTPs. The hydrophobic residues Val, Ala, Met, Leu, Thr, and Tyr are dominant in MTPs and exhibit high- or middle-ranked propensities. All hydrophilic amino acids (Pro, Gln, Asp, Arg, Glu, Asn, Ser) are low-scored and are more favored in non-MTPs

**Table 3:** The MTP propensity score and PCPs selected from AAindex database based on R.

Amino Acid	MTP score (Rank)	KYTJ820101 (Rank)	WERD780104 (Rank)	OLSK800101 (Rank)
I-Ile	571.9 (1)	4,5 (1)	0.06 (5)	2,32 (1)
F-Phe	566.6 (2)	2,8 (4)	0.4 (1)	1,72 (4)
G-Gly	552.8 (3)	-0,4 (8)	0.27 (2)	1,34 (8)
V-Val	526.1 (4)	4,2 (2)	-0.11 (8)	1,99 (2)
A-Ala	521.4 (5)	1,8 (7)	-0.07 (7)	1,38 (7)
M-Met	520.9 (6)	1,9 (6)	0.03 (6)	1,78 (3)
L-Leu	490.2 (7)	3,8 (3)	-0.17 (10)	1,47 (5)
T-Thr	469.7 (8)	-0,7 (9)	0.09 (4)	0,89 (9)
C-Cys	468.5 (9)	2,5 (5)	0.17 (3)	1,43 (6)
Y-Tyr	460.1 (10)	-1,3 (12)	-0.61 (17)	0,47 (16)
S-Ser	433.5 (11)	-0,8 (10)	-0.11 (8)	0,86 (10)
N-Asn	424.7 (12)	-3,5 (15)	-0.57 (16)	0,37 (17)
E-Glu	422.8 (13)	-3,5 (16)	-0.63 (19)	0,71 (13)
R-Arg	415.6 (14)	-4,5 (20)	-0.4 (12)	0 (20)
W-Trp	411.6 (15)	-0,9 (11)	-0.61 (17)	0,82 (12)
D-Asp	407.8 (16)	-3,5 (17)	-0.8 (20)	0,52 (15)
Q-Gln	407.1 (17)	-3,5 (18)	-0.26 (11)	0,22 (18)
H-His	398.4 (18)	-3,2 (14)	-0.49 (15)	0,66 (14)
K-Lys	398.3 (19)	-3,9 (19)	-0.45 (13)	0,15 (19)
P-Pro	396.4 (20)	-1,6 (13)	-0.47 (14)	0,85 (11)
R	1	0.83	0.80	0.86

These observations are consistent with the results from several previous studies. Amongst these, the analysis of AA propensities and physicochemical properties of photosynthetic proteins showed that the hydrophobic interactions are crucial for electron transport reactions [23]. Site-directed mutations of Val102, Phe219, and Glu276 residues are shown to impair the transport function of SmbA protein, which mediates the transport of antimicrobial peptides [32].

The correlation between the propensity scores derived from SCMMTP and amino acid composition is evaluated with the Pearson correlation coefficient (R value). The high correlation coefficient (R = 0.95) between the propensity scores of amino acids and the composition difference between MTPs and non-MTPs indicates that SCMMTP-derived scores are effective in discriminating between positive and negative classes. The distributions of AA propensity scores on the surface of several highly-scored MTPs and non-MTPs have been visualized in Figure 3B. The red color represents the positions of highly-scored amino acids, whereas the low-scored AAs are colored in blue.

As shown in Figure 3, MTPs contain more regions colored in red than non-MTPs. Furthermore, high-scored regions in MTPs are mainly present in the transmembrane α-helices. Hence, the increased occurrence of hydrophobic residues in MTPs, evident from the AA propensity and composition analysis, is due to the presence of long stretches of these residues in the membrane spanning α-helices.

### MTPs characterization using physicochemical properties

In this study, the SCM-PCPs method was used to identify the physicochemical properties (PCPs) of MTPs. The correlations between SCMMTP-derived AA scores and AAindex indices of the PCPs have been estimated and the top-ranked PCPs from AAindex database are presented in Table 3. The three selected PCPs with their corresponding R values are: OLSK800101 or "Average internal preferences" (R = 0.86); KYTJ820101 or "Hydropathy index" (R = 0.85); WERD780104 or "Free energy change of epsilon(i) to alpha(Rh)" (R = 0.74).

#### A. Hydropathiccharacteristics of MTPs

SCM indicates that the KYTJ820101 property, described as the "hydropathy index" [33], was found to have a high positive correlation (R = 0.854) with AA scores. KYTJ820101 represents a hydropathy scale in which each of the 20 amino acids is assigned a value reflecting its relative hydrophobicity and hydrophilicity based on experimental observations [33].

In contrast to soluble proteins, little is understood about the structure and folding of membrane-related proteins. To date, very few high-resolution three-dimensional structures have been solved for membrane proteins due to the need for sophisticated techniques for diffraction studies. Problems originate from the inherent insolubility of membrane proteins due to the presence of hydrophobic domains [34]. In fact, as of mid-February 2012 only 320 unique membrane proteins had been deposited in the protein data bank, representing less than 1% of the total data [35]. While structural determination has progressed in recent years, most membrane protein crystal structures solved are taken from bacteria because eukaryotic membrane proteins are more difficult to crystallize [36].

Given this lack of experimental structural data, three-dimensional structures can be inferred from amino acid sequences applying an appropriate hydrophobicity scale. Examination of the hydropathy of a given sequence can help crystallographers measure the distribution of hydrophobic and hydrophilic regions, predict whether or not a given peptide segment is sufficiently hydrophobic to interact with or reside within the interior of the membrane, define secondary structures, and study the relationships between buried/exposed behaviour of the residues and their nature [33,37].

**Figure 4:**
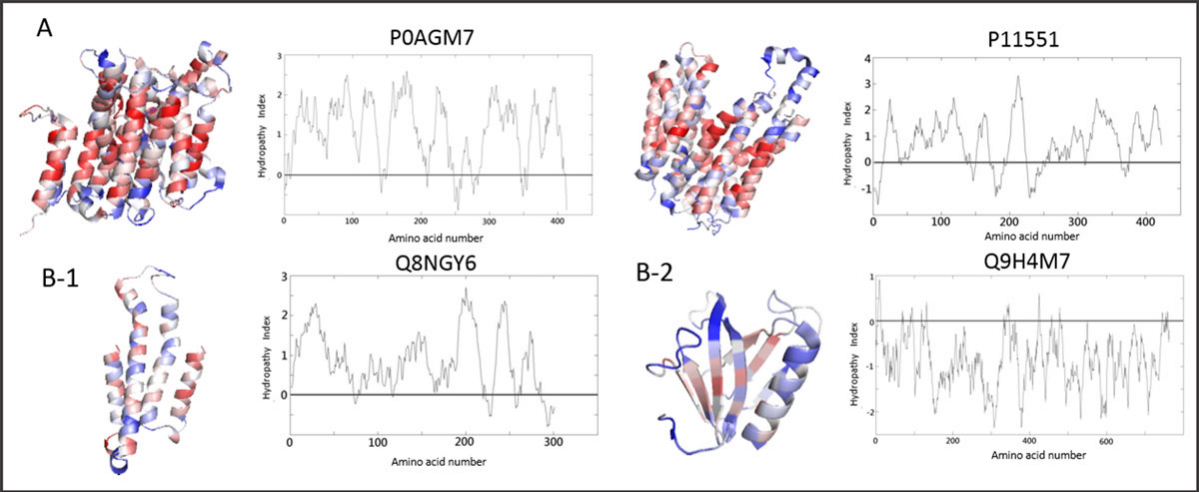
**Amino acid propensity scores visualization and hydropathy plot using hydropathy index**. (A) MTPs (B) Non-MTPs. The red color represents the positions of highly-scored amino acids, whereas the low-scored AAs are colored in blue.

Many hydrophobicity scales have already been developed, but the question remains: Which scale, if any, reflects the tendency of MTPs to best adopt their conformation and associate with membranes? Results from the present study indicate that a combined scale, formulated by Kate & Doolittle [33] is the most appropriate for the hydropathy analysis of the membrane transporter proteins included in our datasets, as well as those predicted by SCM. Figure 4 provides an example of hydropathy plots of MTPs and non-MTPs.

Figure 4A shows hydropathy plots ofthe MTPs, P0AGM7 and P11551. It clearly shows the physicochemical property of "surrounding hydrophobicity in α -helix", which has a high positive correlation (R = 0.836) with AA scores. In Figure 4B-1, the non-MTP Q8NGY6 showed the transmembrane regions but displayed few high-scoring amino acids in the α-helix. In Figure 4B-2, the non-MTP Q9H4M7does not show the transmembrane regions, thus Figure 4 indicates these two PCPs are important classification features.

Furthermore, the correlation results obtained between SCM scores and the proposed hydropathy values [33] reveal that Ile plays a significant role in the stability and functionality of MTPs. Ile, Phe, Gly, Ala and Val residues ranked top-5 in our SCM derived scale are hydrophobic and are top- or middle-ranked in the proposed hydropathy scale [33]. Further investigation is needed to determine its role in the structure and stability of membrane transporter proteins.

Hydrophilic Arg, Gln, Asp, Lys and Glu residues are respectively ranked by Kate & Doolittle [33] as the bottom-20, -18, -15, -19 and -16, are also at the bottom-5 of SCM derived scores. This may imply reduce prevalence of hydrophilic residues in membrane transporter protein native structures.

The abundance of Ile and Phe residues in the membrane proteins agrees with previous findings, which mention their location in the acyl chain areas of membrane lipids [36]. It should also be noted that steric effects may affect the folding of membrane transporter proteins independent of hydropathy [33].

#### B. Preferences of transporters to form outside α-helices

The WERD780104 property showed a high correlation (R = 0.7445) with SCM-derived scores and is described in the AAindex as "free energy change of epsilon(i) to alpha(Rh)" [38]. The amino acid indices of WERD780104 reflect the effects of local solute-solvent interactions (i.e., interactions between a residue and water with no influence from the neighboring residues) on the conformational preferences of the 20 naturally occurring amino acids, summarized from protein X-ray data. In a given property [11], different residue conformations have been assigned to one of the three types of protein backbone structures: nonregular structure, helix, and extended structure. Nonregular structures include residues in an epsilon(i) conformation, which defines isolated extended residues or those which are a part of a run of two or three extended residues. On the other hand, the helix structure included residues in α_Rh _conformation, defining those residues as a part of the right-handed α-helix. In WERD780104, the free energy change (Δ(ΔG°)) was used to express the preferences of each residue for a nonregular structure relative to their preferences for the α-helical structure in going from the inside to the outside of a protein molecule.

In general, the scale proposed by Wertz et al. [38] confirmed the general preference of polar groups to be on the outside of protein molecules, while the non-polar groups are on the inside. However, regarding the conformational pattern, important inferences can be drawn from the obtained positive correlation results between SCM scores and the WERD780104 scale of free energy changes as follows: 1) transporter proteins are more stable in α-helical structures than in non-regular or extended structures and 2) transporter proteins have higher preferences for α-helices outside, rather than inside.

Molecular transport proteins are regarded as 'outside' or surface polypeptide chains and face the cavity, pore or channel, in contrast to membrane-buried regions. As discussed by Wertz et al. [38], proteins are usually more stable if they have non-regular or helical structures on the surface, because of the greater increase in entropy in going from the inside (where the librational motions of all types of residues are highly restricted) to the outside of a protein (where the restrictions on the librational motions are less severe).

### Characterization MTP using both the propensity visualization and physicochemical properties

**Figure 5:**
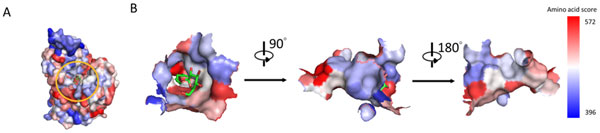
**The surface heat-map of fucose transporter (PDB:3O7P)**. A. the surface heat-map of fucose transporter. The surface contacting with the ligand presents the light blue, light red and white according to amino acid propensity scores. The ligand is presented as the sticks. The orange circle indicates the binding site. B. Heat map of the binding site surface in top-view and the views of different rotations.

The training dataset, MTP-TRN130, contains 260 cation transporters, which constitute the most of the MTPs, in contrast to the amino acid, anion, electron, protein/mRNA, sugar and other transporters which contain 70, 60, 60, 70, 60 and 200 sequences, respectively. Therefore, the characteristics of the cation transporters would dominate the characteristics of other MTPsdescribed above. MTPs have an "inside-out" property [39] causing the exposure of hydrophobic residues on the surfaces that face the membrane environment. The channels which transporthydrophilic molecules would be composed of hydrophilic residues to reduce free energy barrier during the transporting process. The surface heat-maps generated using propensity visualization indicates these residues are not particularly hydrophilic ones, with low-scoring residues shown in blue in Figure 5.

Amino acid compositions of the transmembrane segments are used to investigate the relationship of the propensity scores and transmembrane segmentfor channel location, the amino acid compositions. Previous studies [40,41] investigated the multi-span and single-span transmembrane segment amino acid compositions. As illustrated in Table 4 the propensity scores show respective correlations of 0.78 and 0.82 to multi-span helix AACs and single span helix AACs, suggesting that these propensity scores are closely related to the transmembrane segments.

**Table 4:** The correlations between the propensity scores and PCPs including membrane single span helix, membrane multi-span helix and amino acid hydration energies.

Amino acid	score card	**AAC of single span segment **[40]	NAKH920108	WOLR810101	**Hydration energy**[43].
I-Ile	571.9	3.46	13.73	2.15	-10.9
F-Phe	566.6	1.48	10.99	-0.76	-12.3
G-Gly	552.8	1.27	6.17	2.39	-14.5
V-Val	526.1	2.46	12.43	1.99	-11.6
A-Ala	521.4	1.73	9.36	1.94	-12
M-Met	520.9	0.86	3.93	-1.48	-12.5
L-Leu	490.2	2.56	16.64	2.28	-11.3
T-Thr	469.7	0.59	4.68	-4.88	-13.6
C-Cys	468.5	0.84	2.56	-1.24	-13.1
Y-Tyr	460.1	0.59	3.13	-6.11	-16.9
S-Ser	433.5	0.49	5.58	-5.06	-14.8
N-Asn	424.7	0.01	2.31	-9.68	-17.5
E-Glu	422.8	0.01	0.94	-10.2	NA
R-Arg	415.6	0	0.27	-19.92	NA
W-Trp	411.6	0.74	2.2	-5.88	-15.2
D-Asp	407.8	0.03	0.94	-10.95	NA
Q-Gln	407.1	0.03	1.14	-9.38	-17.9
H-His	398.4	0.06	0.47	-10.27	-20
K-Lys	398.3	0.03	0.58	-9.52	NA
P-Pro	396.4	0.18	1.96	-3.68	NA

R1^a^	1	0.82	0.78	0.79	0.79
R2^b^	1	0.87	0.77	0.54	0.80

^a ^the correlation between the propensity scores of all residues and the PCPs

^b ^the correlation between the propensity score of polar residues and the PCPs

The polar residues are thought to play an important role in ion selecting, depending on their hydration energy. Illergard et al. [42] indicated the polar residues in the core of membrane proteins are conserved and often interact with water. Thissuggests that the polar residues of transmembrane proteins usually work in aqueous environments and the hydration energy influences the channel selectivity. The hydration energy of each amino acid is also provided in Table 4. WOLR810101 is the "hydration potential" provided from the AAindex, and a newer hydration potentials are also provided from Konig et al. [43]. The high correlations (0.79) between the propensity scores and the hydration energies indicate the transmembrane segments are prone to be composed of low hydration energy residues. The MTPs are folded in the membranes, which is an extremely hydrophobic environment, and are composed of the hydrophobic low hydration energy amino acids that could decrease the folding energy [44]. Since polar residues are important to ion selection, the relationship of the polar residue propensity scores to the hydration energies of polar residues is also shown in Table 4.

The correlations of amino acid scores between WOLR810101 and hydration energy are 0.54 and 0.80, respectively, suggests that the correlation increases after updating the hydration energies. It also indicates that the high hydration energy residues have high propensity scores. This leads to the conclusion that the transmembrane regions are prone to being composed of high hydration energy amino acids, and the polar residues in the transmembrane regions are responsible for transporting hydrophilic molecules.

**Figure 6:**
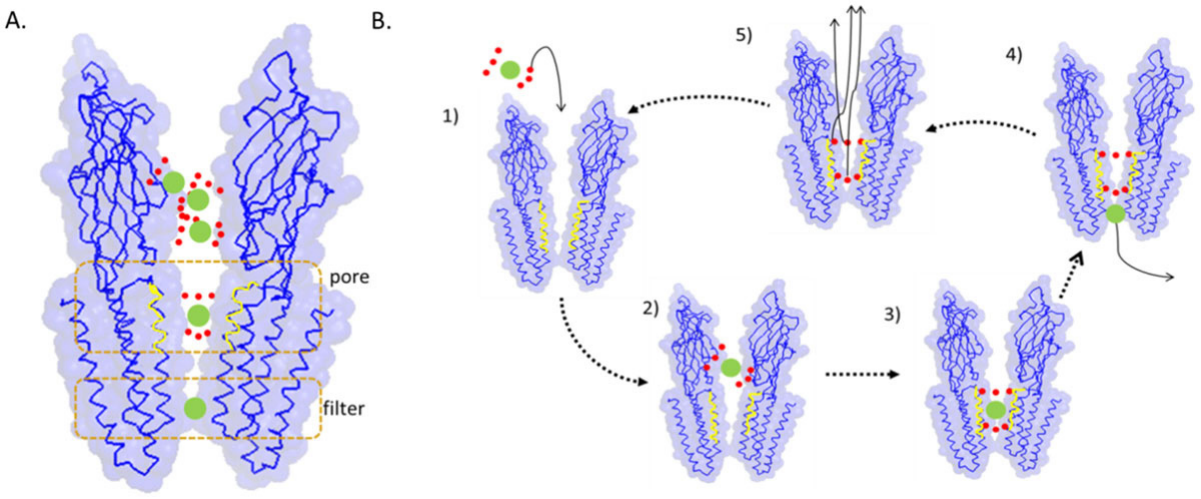
**The example of ion channel and the ion filter process**. The channel protein modified from pentameric ligand gated ion channel (PDBID:3EHZ) is presented using Pymol. A. the protein is shown in ribbon and colored in blue and the yellow lines denote the polar residues composing of the pore region. The greens dots indicate the ions and the red dots mean water molecules. The orang dash squares indicate the pore and filter regions. B. the proposed ion selecting mechanisms: 1) ion are shelled with and the channel do not contain ions; 2) the ion with water shell enter into the channel; 3) the water shelling the ion will be fixed by the polar residues using hydrogen bonds; 4) after the coordination of the ion and water molecules are fix, the ion can easy go through the filter region and is stabilized with the filter residues; 5) the water molecules composing empty shell will release out from the pore region and the channel change to the empty state for next ions.

The filter mechanisms of channel proteins are investigated to provide insights into the selectivity of membrane transporter proteins. As shown in Figure 6, the channel proteins usually have filter and pore regions [11]. Ion selection in the filter region depends on the ion hydration states, while the pore region adjusts the ion coordination [13]. The hypothetical filter steps are provided in Figure 6B. Ions have a water shell in water solution. After ions enter the channel pores(which are often composed of polar residues) the water around the ions will be fixed to the polar residues by hydrogen bonds. The ions escape from the water shell using the side chains of the filter residue, which can stabilize the ion in a dehydration state [12,13]. Once the ion escapes from the water shell and crosses the membrane, the water molecules should leave the pore region to fix the next ions. If the polar residues composing the pore regions have low hydration energies, water molecules cannot easily escape from the pore region, thus decreasing ion channel efficiency.

## Conclusions

Despite the growing amount of sequences of MTPs in public databases, their three-dimensional structures are being resolved at far more slower rates. In this study, several machine learning methods have been applied to predict MTPs from sequences. Furthermore, a novel scoring card (SCM)-based SCMMTP method have been proposed for prediction and analysis of MTPs. SCMMTP method yielded a good prediction performance and utilized dipeptide and amino acid propensity scores of MTPs to analyze their structure and physicochemical properties.

Considering the importance of MTPs in numerous biological processes, understanding of their nature can help to facilitate important future applications, including drug design.

## Competing interests

The authors declare that they have no competing interests.

## Authors' contributions

HLH, TV and YFL participated in manuscript preparation. TV, YFL and CLYanalyzed the physicochemical properties and protein visualization. PC, LSH and WCL implemented the programs. WCL, YFL established the website. SHC manipulated the materials. HLH and SYH participated in the system design, supervised the whole project and coordination, and helped to write the manuscript. All authors have read and approved the final manuscript.

## Supplementary Material

Additional file 1 **Table S1**. Performance comparison of MTP predictors on the training set. Click here for file

Additional file 2 **Table S2**. 10 independent runs of SCMMTP on the training set. Click here for file

Additional file 3 **Table S3**. Amino acid propensity scores and composition in MTP and non-MTP. Click here for file

## Declarations

This work was also funded by Ministry of Science and Technology of Taiwan under the contract number MOST-104-2627-M-009-009-, MOST-104-2221-E-009-183-, and "Center for Bioinformatics Research of Aiming for the Top University Program" of the National Chiao Tung University and Ministry of Education, Taiwan, R.O.C. for the project 104W962. This work was also supported in part by the UST-UCSDInternational Center of Excellence in Advanced Bioengineering sponsored bythe Taiwan Ministry of Science and Technology with I-RiCE Program under Grant Number:MOST-103-2911-I-009-101-

This article has been published as part of *BMC Genomics *Volume 16 Supplement 12, 2015: Joint 26th Genome Informatics Workshop and 14th International Conference on Bioinformatics: Genomics. The full contents of the supplement are available online at http://www.biomedcentral.com/bmcgenomics/supplements/16/S12.
