# Supplementary material for: SCMMTP: identifying and characterizing membrane transport proteins using propensity scores of dipeptides
Source: BMC Genomics. 2015 Dec 9;16(Suppl 12):S6. doi: 10.1186/1471-2164-16-S12-S6 (PMC4682407; doi:10.1186/1471-2164-16-S12-S6)
Supplement: Additional file 1 — Table S1. Performance comparison of MTP predictors on the training set. [file 1471-2164-16-S12-S6-S1.pdf]

Table S1 - Performance comparison of MTP predictors on the training set

Method	Acc (%)	Sensitivity (%)	Specificity (%)	MCC
**Bayes-AAC**	69.30	74.87	62.04	0.37
**Bayes-DPC**	71.04	78.33	61.54	0.41
**Bayes-AAindex**	67.20	73.46	59.03	0.33
**Bayes-PSSM**	73.88	85.77	58.36	0.46
**J48-AAC**	68.00	72.56	62.04	0.35
**J48-DPC**	62.26	67.56	55.35	0.23
**J48-AAindex**	67.49	69.23	65.22	0.34
**J48-PSSM**	69.88	74.62	63.71	0.38
**KNN-AAC(k=7)**	70.54	86.28	50.00	0.39
**KNN-DPC(k=5)**	64.08	87.82	33.11	0.25
**KNN-AAindex(k=7)**	71.99	79.87	61.71	0.42
**KNN-PSSM(k=13)**	73.88	85.77	58.36	0.46
**SVM-AAC**	75.69	78.64	71.88	0.51
**SVM-DPC**	72.71	75.44	69.01	0.44
**SVM-AAindex**	76.27	78.10	72.28	0.50
**SVM-PSSM**	76.63	80.32	72.30	0.53
**SCMMTP**	81.12	83.76	77.68	0.62
**Mean**	71.29	78.38	61.98	0.41
