# Supplementary material for: SCMMTP: identifying and characterizing membrane transport proteins using propensity scores of dipeptides
Source: BMC Genomics. 2015 Dec 9;16(Suppl 12):S6. doi: 10.1186/1471-2164-16-S12-S6 (PMC4682407; doi:10.1186/1471-2164-16-S12-S6)
Supplement: Additional file 2 — Table S2. 10 independent runs of SCMMTP on the training set. [file 1471-2164-16-S12-S6-S2.pdf]

Table S2. 10 independent runs of SCMMTP on the training set

Exp.	Fitness Score	Train Acc (%)	Sensitivity (%)	Specificity (%)	Threshold
**1**	0.8899	81.71	0.85	0.78	476.88
**2**	0.8880	81.64	0.83	0.80	486.71
**3**	0.8871	80.70	0.83	0.78	497.11
**4**	0.8867	81.20	0.81	0.81	504.76
**5**	0.8860	81.06	0.84	0.77	475.41
**6**	0.8854	81.28	0.84	0.77	497.57
**7**	0.8851	80.55	0.86	0.74	481.35
**8**	0.8849	80.62	0.80	0.82	497.82
**9**	0.8849	81.28	0.85	0.77	498.12
**10**	0.8836	81.13	0.87	0.74	494.12
**Mean**	0.8861	81.12	0.84	0.78	490.98
**STDV**	0.0017	0.38	0.02	0.03	9.65
