# Supplementary material for: SCMMTP: identifying and characterizing membrane transport proteins using propensity scores of dipeptides
Source: BMC Genomics. 2015 Dec 9;16(Suppl 12):S6. doi: 10.1186/1471-2164-16-S12-S6 (PMC4682407; doi:10.1186/1471-2164-16-S12-S6)
Supplement: Additional file 3 — Table S3. Amino acid propensity scores and composition in MTP and non-MTP. [file 1471-2164-16-S12-S6-S3.pdf]

Table S3. Amino acid propensity scores and composition in MTP and non-MTP

Amino acid	MTP propensity Score (Rank)	Composition of MTP: A (%)	Composition of non-MTP: B (%)	Composition difference: A-B (%)
**I-Ile**	571.9 (1)	6.64	5.40	1.24
**F-Phe**	566.6 (2)	5.51	4.43	1.08
**G-Gly**	552.8 (3)	7.03	6.00	1.03
**V-Val**	526.1 (4)	7.11	6.30	0.81
**A-Ala**	521.4 (5)	7.82	7.00	0.81
**M-Met**	520.9 (6)	2.79	2.29	0.50
**L-Leu**	490.2 (7)	11.08	10.55	0.53
**T-Thr**	469.7 (8)	5.53	5.36	0.17
**C-Cys**	468.5 (9)	1.61	1.65	-0.04
**Y-Tyr**	460.1 (10)	3.19	3.17	0.02
**S-Ser**	433.5 (11)	7.69	8.28	-0.59
**N-Asn**	424.7 (12)	3.87	4.27	-0.40
**E-Glu**	422.8 (13)	5.19	6.15	-0.96
**R-Arg**	415.6 (14)	4.53	5.19	-0.66
**W-Trp**	411.6 (15)	1.50	1.53	-0.03
**D-Asp**	407.8 (16)	4.15	4.89	-0.75
**Q-Gln**	407.1 (17)	3.57	4.29	-0.72
**H-His**	398.4 (18)	1.89	2.37	-0.48
**K-Lys**	398.3 (19)	4.78	5.65	-0.87
**P-Pro**	396.4 (20)	4.54	5.23	-0.68
**R**	1.00	0.46	0.18	0.95
